# Carboxymethyl cellulose/shellac composite loaded with pomegranate extract and jojoba oil as anti-mycotic and anti-mycotoxigenic food packaging materials

**DOI:** 10.1038/s41598-024-81933-7

**Published:** 2025-01-06

**Authors:** Salah A. A. Mohamed, Amr Farouk, Adel G. Abdel-Razek, El-Shahat Nashy, Mohamed El-Sakhawy, Ahmed Noah Badr

**Affiliations:** 1 Packing and Packaging Materials Department, National Research Centre, Dokki, Cairo, 12622 Egypt; 2 Chemistry of Flavor and Aroma Department, National Research Centre, Dokki, Cairo, 12622 Egypt; 3 Fats and Oils Department, National Research Centre, Dokki, Cairo, 12622 Egypt; 4 Chemical Industries Research Institute, National Research Centre, Dokki, Cairo, 12622 Egypt; 5 Cellulose & Paper Department, National Research Centre, Dokki, Cairo, 12622 Egypt; 6 Food Toxicology and Contaminants Department, National Research Centre, Dokki, Cairo, 12622 Egypt

**Keywords:** Edible composite, Pomegranate, Jojoba oil, Anti-mycotoxigenic, Packaging food, Molecular docking, Microbiology, Plant sciences

## Abstract

Food commodities, including mycotoxins naturally produced from toxigenic fungi (pre- or post-harvest), are particularly vulnerable to contamination. The study intended to use unique bioactive composites loaded with antimicrobial constituents for food packaging. Three composite types are based on carboxymethyl cellulose/shellac (CMC/SH) and loaded with pomegranate extract (POE) with or without jojoba oil (JOE) at various concentrations. An enhancement was recorded for tensile strength and elongation at break and burst properties of the composites, where the results point out the amelioration of flexibility and elasticity with E9 (0.3/3 mg/mL of POE/JO). Moreover, E10 (0.3/1 of POE/JOE) content had higher phenolic and flavonoids, with significant antioxidants and the best antimicrobial and anti-mycotoxigenic activity. Six higher antimicrobial composites were chosen for corn seed coating applications in a simulated experiment of toxigenic fungal contamination, where the results recommend E10 as the best formula for packaging application. The E10 was characterized for emulsion stability, particle size, zeta potential, pH, PDI, and acidity that were recorded at 88.16 ± 2.87%, 54.81 nm, 38.74 mV, 6.34 ± 0.54, 31.12 ± 1.02, and 6.02 ± 0.34 mg/L, respectively. The *in-silico* study revealed that ellagic acid and hesperidin in POE extract, erucic and oleic acids in JOE, and shellac had the highest binding free energies against the vital enzymes involved in bactericidal/bacteriostatic effects and the aflatoxin bio synthetic mechanism.

## Introduction

Food is produced with various raw, semi-processed, and final products. Food materials could be passed through handling stages, from pre-harvesting, like cereals, grains, and horticultural crops, to manufactured food. Food products may suffer from spoilage and contamination during transportation, storage, and processing^1^. Two significant points for human food are food safety and security. Food safety is related to being free of contamination and spoilage factors, while food security is included in availability^2^. The high occurrence of contamination could lead to food losses, which results in food insecurity and health issues^3,4^. In this regard, increasing the safety conditions of food materials through the stages before consumption is essential.

Toxigenic fungi are considered a high-risk factor leading to food losses and show non-safe foods^5^. *Aspergillus flavus* and *A. parasiticus* are the most common fungi that generate toxic metabolites in warm and humid climates such as the Mediterranean basin^6,7^. These fungi can produce secondary metabolites, causing public health issues known as mycotoxins. Aflatoxins are a group of mycotoxins considered the most hazardous metabolites contaminating food, including cereals, during processing^8,9^. Bioactive components like antioxidants, phenolics, flavonoids, and waxy materials can support fungal inhibition and reduce aflatoxin accumulation in food products^10^. Food byproduct extracts are a rich source of such bioactive components. Applying these materials in food processing can limit the development of toxigenic fungi and mycotoxins^11^.

Bacterial pathogens were reported to contaminate food materials, and the packaging was deemed a suitable solution against that. It is widely recognized that packaging’s principal function is to protect food products against pathogens, moisture, pollutants, odors, and mechanical damage^12^. Antimicrobial packaging is created by applying an antimicrobial substance to polymeric packaging composites. Antimicrobial substances can migrate to the food surface or prevent bacteria growth on the food surface without migrating^13^. Antimicrobial packaging is an innovative advancement integrating natural or chemical preservatives into a composite film to limit pathogen activity^14^. Nevertheless, antimicrobial packaging development remains a complex technology, with limited available commercial products. Polymeric film materials can protect food materials against several pathogens, including *Staphylococcus, Bacillus****,*** and *Pseudomonas*^15,16^. Otherwise, food composite film loaded using natural extract possessed antifungal activity against toxigenic fungi, including *Aspergillus flavus, A. parasiticus*^17^, and other mycotoxigenic strains of fungi^18^.

Pomegranate byproducts, such as peels and seeds, are produced during fruit processing, where they contain abundant bioactive substances, including phenolic compounds, dietary fiber, alkaloids, minerals, and vitamins with antioxidants and extensive anti-inflammatory, anti-cancer, antibacterial, and cardiovascular protection functions. After extraction, it can be incorporated into various food products to preserve their ‘clean label’ status and enhance their functional properties^19,20^. Besides, jojoba oil was reported to have antifungal and anti-mycotoxigenic effects. Combining jojoba oil (carrier) and pomegranate extract (bioactive component source) may enhance their anti-aflatoxigenic impact^21^. Moreover, nano and micro-emulsions can be utilized as a distinguished technique to carry active extracts and assess the limitation of aflatoxin contamination^22^.

The process of forming food films in the form of composites is considered one of the promising technologies that have a good effect on preserving food from spoilage causes, whether related to contamination with harmful toxigenic fungi or mycotoxins^23–25^. A composite constructed using natural components is a temporary package material to enhance food safety and avoid contamination^26^. Composites can be created using an assisted technique such as nanoemulsion, which could be utilized to valorize food safety from farm to fork^27^. Changes applied to the ratios of the components in the composite will have varying properties and characteristic impacts on the formed coating composite. Furthermore, the composite that supports rapid drying of the composite enhances its functionality, efficiency, and capacity. Also, loading the active substances onto the composite compound allows the control-releasing of active substances during handling and storage^28^.

Shellac, HPMC, and graphene oxide are environmentally friendly polymers and nano-composites^29^. In particular, carboxymethyl cellulose (CMC) has excellent film-forming characteristics with a water-soluble polymer and heat gelatinization^30^. However, cellulose derivative films offer inadequate water vapor barriers because they are hydrophilic^31^. The Food and Drug Administration (FDA) classified a natural polymer, shellac, as a safe food additive in coating films^32^. It is a low-molecular-weight resin mostly made up of oxyacid polyesters^33^. Recently, a composite of gelatin, shellac, and the CMC with minimal porosity, high homogeneity, and antimicrobial qualities was created. This composite tended to particle aggregation, high homogeneity, tensile strength, elongation, and air permeability^34^. These polymer applications in food film composite formation varied according to the purpose of their utilization.

Natural polymer composites of the CMC and shellac loaded with pomegranate and jojoba extracts could be used as safe packaging materials instead of synthetic films. The work objective was to use compatible natural extracts loaded into film composite as unique bio-food packaging material. The present study compared emulsion composites to form a unique food composite film with antifungal and anti-mycotoxigenic potency. Selected types of prepared composite ability were examined in fungal media to limit aflatoxin secretion; they also investigated in a simulated experiment using inoculated corn (with/without coating) to evaluate real composite efficiency as food packaging.

## Materials and methods

### Raw materials, chemicals, and microbial strains

The standard of aflatoxin B_1_ (AFB_1_) was obtained from Sigma-Aldrich, Saint Louis, MO, USA. Ascorbic acid, Gallic acid, DPPH, and catechol were purchased from Sigma Aldrich and were analytical grade, and utilized solvents were HPLC grade. De-ionized water was applied in water extraction. The UV-spectrophotometer, Shimadzu UV-1201 model, was used for colorimetric analysis.

The Gram-positive (*Bacillus cereus* EMCC 1080, *Staphylococcus aureus* ATCC 13,565) and the Gram-negative (*Salmonella typhi* ATCC 15,566; *Pseudomonas aeruginosa* NRRL B-272) bacteria were utilized for the determination of antibacterial effect. These isolates were received from the DSMZ microbial collection (Leibniz Institute DSMZ-German Collection of Microorganisms and Cell Cultures, Braunschweig, Germany), maintained on nutrient agar slants for 24 h/ 37 °C, and kept in the refrigerator (4°C) until use. These strains were selected as the most dominant pathogens of Gram-positive and Gram–negative bacteria that affected food materials during storage and led to food-borne illnesses.

The antifungal experiment was done on four strains of toxigenic fungi obtained from the agro-food microbial culture collection (ITEM), ISPA, CNR, Italy. *Aspergillus flavus* ITEM 698, *A. niger* ITEM 7097, *Penicillium verrucosum* NRRL 695, and *Fusarium graminearum* ATCC 56,091. Before the assessment test, fungal strains were preserved on Czapek-dox media. For the simulated antifungal experiment, strains of *A. parasiticus* ITEM 11, *Fusarium culmorum* KF191, and Alternaria alternate sp. were applied in liquid media as mycotoxin-producing fungi. The strains mentioned above of fungi belong to toxigenic fungi that produce toxins in food products, which cause a dowel source of contamination (fungal growth and toxin production. Applied strains of bacterial pathogens and toxigenic fungi used in the present study were utilized to evaluate composite film types as a new trend in food packaging materials. All chemicals, standards, and media were purchased from Sigma-Aldrich Chemical Co. (St. Louis, MO, US), where solvents and chemicals were of analytical chromatographic grade.

### Raw materials extraction

The active natural raw material of pomegranate peel extract (POE) was prepared using aqueous isopropyl (80%) as an eco-friendly system, and the extraction efficiency was enhanced using an Ultrasonic probe (amplitude 45%, 80 kHz, duty 60%, time 40 min, 20℃). The POE consisted of rinds, peels, and seeds as a natural ratio of existence in the fruit. The collected extract was lyophilized (Laboratory Lyophilizer, FD-10-MRMalti-manifold, Esquire Biotech, India) and kept cool until further evaluations. Jojoba crude oil (JOE) was extracted from fresh seeds using a cold press system consisting of a Manual hydraulic press (160 kg/cm^2^/ 30 min) with perforated stainless-steel trays (30 cm diameter X 10 Stacked trays). The seeds were piled onto trays, where the oil was liquefied into a basin. The collected oil was preserved cool in an amber bottle.

### Determination of fatty acids composition

Fatty acids methyl esters (FAMEs) were prepared according to the AOCS Official Method Ce 1k‐07^35^. Diluted FAMEs were separated on an HP 5890 series II (Hewlett-Packard, Palo Alto, USA) equipped with FID and Innowax column (30 m*0.20 mm*0.20 µm) at a 1.5mL H_2_/min flow rate. The isotherm temperature of the column and the detector were adjusted at 210°C and 240°C, respectively. The fatty acid percentages were identified using the rapprochements with the authentic standards retention times.

### Preparation of several composite formulas

Based on previous work, CMC/Shellac composites were reported to have better elasticity, air permeability, and tensile strength^34^. Forward to that, it was chosen as a base composite to formulate a distinguish-loaded nano-composite for food packaging. The composites were prepared using a variation between the ingredients’ ratios of base (CMC/SH) to choose a more suitable base for lading (E1-E4). A solution of CMC was prepared using double distilled water at a concentration of (2%). Shellac solution was dissolved in absolute ethyl alcohol at (5%) concentration. Tween 80 and glycerol were applied as emulsifiers and surfactant agents for stabilizing impact; ethanol was used to dissolve the POE-concentrated powder and as a co-surfactant. Sixteen emulsion combinations were ready for evaluation according to the ingredient amounts represented in Table 1.

**Table 1:** composite formulas preparation and their ingredients.

	Controls				Loaded composites for evaluations											
	E1	E2	E3	E4	E5	E6	E7	E8	E9	E10	E11	E12	E13	E14	E15	E16
CMC (g)	0.84	0.12	0.84	-	0.84										0.9	1.05
Shellac (g)	0.3	2.1	-	2.1	0.3											
JOE (mL)	-	-	3		3					1	2.5	5	7.5	10	1	1
POE (mg/mL)	-	-	0.3		-	0.075	0.15	0.225	0.3	0.3						

Emulsions from the CMC and shellac (60 mL solution volume, completed with glycerol) were loaded by the bioactive components (JOE; POE) at different concentrations to compare the efficiency regarding their concentrations. However, in E15 and E16, the CMC concentration was increased to enhance stability and provide good droplet size uniformity^36^. To form the dry shape of composites, they were cast from aqueous suspensions in Petri dishes and dried (40 ℃/12 h) using the hot–air oven with circulating air.

### Characterization of emulsion composites

Tensile strength, burst strength, air permeability test, thickness, and scanning electron microscope were determined to characterize the formed composites. Tensile measurements were carried out with a Lloyd instrument (Lloyd Instruments, West Sussex, United Kingdom) with a 100-N load cell. The measurements were performed on strips with 1.5 cm width and 8 cm length at a crosshead speed of 2 mm/min at 25 °C. Three replicates of each sample were measured, and the results were averaged.

Burst strength was carried out according to TAPPI (Standard test method T 403 om-97) using Mullen (Perkins, Chicopee, MA, USA). The Air permeability test was conducted on the BENDTSEN Smoothness and Porosity Tester, Model 5, No. 11772, Andersson and Sorensen, Copenhagen. The thickness of the specimens was measured using a Mitutoyo "Absolute Aos Digimatic" measuring range of 0-150 mm, cod no. 500–181-30 model no. CD-15APX; the average of five measurements were taken. For Scanning electron microscope examination (SEM), Samples were subjected to sputter coating (Edwards’ model S 140A) of gold ions to have a conducting medium. Sputter-coated samples were scanned with SEM- JEOL Model JSM-T20.

### Determination of antioxidant scavenging activity

#### Total phenolic content (TPC)

The total phenolic content of the POE, the JOE, and the prepared composites composites was evaluated using the Folin-Ciocalteu method^37^**.** In contrast, about 125μL of Folin-Ciocalteu reagent (0.2 N) was combined with 25μL of the estimated sample and 100μL of sodium carbonate solution (7.5% w/v). The solution was kept in the dark (2 h/25℃), and then absorbance was measured (at 760 nm) against the blank. A Gallic acid calibration curve (0.01–1 mg/mL) was employed to express the TPC of the examined samples. The findings were expressed as Gallic acid equivalents (mg GAE/100 g dry weight).

#### Total flavonoid content (TFC)

The total flavonoid content of the investigated samples (TFC) was estimated spectrophotometrically following the methodology described previously^38^**.** The assay considered the aluminum chloride colorimetric assay and used rutin as a standard reference. All samples were examined in triplicates. A precise amount of 1 mL of investigated samples was added to 0.3 mL of NaNO_2_ (5%) solution, and after 5 min, a volume of 0.3 mL of AlCl_3_ (10%) was added. Finally, 2 mL NaOH (1M), followed by water, was added to 10 mL. The instantaneous absorbance was measured (at 510 nm), where the results were expressed as rutin equivalents (RE) (mg RE/100 g dry weight).

#### DPPH scavenging activity assay

The effect of the extract on DPPH (1,1 di-phenyl 2-picrylhydrazyl) scavenging was estimated according to Brand–Williams et al.^39^. Absorbance was measured at 515 nm, taking ascorbic acid as the reference**.** Results expressed as inhibition ratio in radical scavenging activity after one hour and 24 h. The antioxidant capacity of the extract solution was estimated using the following formula in Eq. (1):

1 $$\text{\%}{\varvec{T}}{\varvec{A}}{\varvec{C}} = [({\varvec{c}}{\varvec{o}}{\varvec{n}}{\varvec{t}} abs - {\varvec{s}}{\varvec{a}}{\varvec{m}}{\varvec{p}} abs)/ ({\varvec{c}}{\varvec{o}}{\varvec{n}}{\varvec{t}} abs)] x 100.$$

**Where** abs: represents the absorbance value.TAC: Total antioxidant capacityCont: the control.Samp: evaluated sample

#### ABTS free radical scavenging assay

This assay relies on the antioxidant ability of the samples to inhibit the oxidation of ABTS to ABTS^±^ radical cation as a described method used by Arnao et al.^40^**.** The modified auto-bleaching technique was used for the radical scavenging activity of the ABTS [2, 2′-Azino-bis (3-ethyl benzothiazoline-6-sulfonic acid) di-ammonium salt] test**.** Briefly, A 7 mM concentration of 2, 2′-azino-bis (3-ethylbenzothiazoline-6-sulphonic acid) (ABTS) was dissolved in water. The ABTS radical cation (ABTS^**±**^) was generated by combining ABTS stock solution with 2.45 mM K₂S₂O₈, and the solution was kept to rest in the dark (12–16 h) before use. The ABTS ± solution was diluted with water to (0.70 ± 0.05), where the absorbance was measured at 734 nm. A quantity of 0.07 mL of examined samples and 3 mL of the ABTS radical were used in the reaction. After six minutes of incubation, absorbance at 734 nm was measured (using a Shimadzu UV-1201 spectrophotometer). The antioxidant activity was estimated using the Eq. (2)

2 $$\text{\%}TAC = [({\varvec{c}}{\varvec{o}}{\varvec{n}}{\varvec{t}} abs - {\varvec{s}}{\varvec{a}}{\varvec{m}}{\varvec{p}} abs)/ ({\varvec{c}}{\varvec{o}}{\varvec{n}}{\varvec{t}} abs)] x 100.$$

Where abs: represents the absorbance value.TAC: Total antioxidant capacityCont: the control.Samp: evaluated sampleabs: absorbance

### Determination of emulsion characteristics

The composite in emulsion form with the most potential antimicrobial and anti-mycotoxigenic properties was subjected to physicochemical examination. The prepared emulsion’s Zeta potential, particle size, and poly dispersing index were evaluated according to the methodology described before^41^. In brief, Nano-Sizer equipment (Nano-S90, Zetasizer, Malvern Panalytical Ltd, Enigma Business Park, Grovewood Road, United Kingdom)) was utilized to determine the emulsion properties.

Viscosity, pH, and acidity were also determined for the prepared emulsion using the methodology described by Farouk et al.^42^. The determination of the emulsion stability index is based on the separation of the serum from the emulsion. The percentage of separation of the emulsion may be determined by monitoring the height of the separated serum according to Surh et al.^43^, as calculated using the provided Eq. (3)

3 $$\% {\varvec{S}}{\varvec{E}} =({\varvec{H}}{\varvec{u}}/{\varvec{H}}{\varvec{i}})\times 100$$

Where *H*u: is separated upper phase height.*H*i: represents the initial emulsion height. *SE*: the stability ratio for the formed emulsion.

A model of each composite type (CMC; Shellac; base composite CMC/SH; loaded composite of CMC/SH- POE/JOE, were checked for their active sites. Composite was scanned using FTIR spectrometer Spectrum 100 (Perkin Elmer, USA) equipped with an attenuated total reflectance (ATR) module. The sample scanning frequencies were in a range of 4000 to 650 cm^-1^ collected in 32 scans with a spectra resolution of 4 cm^-1^. Measurements were performed at 25 °C, and data were collected in triplicate.

### Antimicrobial activity

#### Antibacterial activity

The antimicrobial effect of raw and emulsion-inserted materials was determined using antibacterial assay according to the technique following the recommendations of the Clinical and Laboratory Standard Institute^44^**.** Examined materials were tested for their agar-well diffusion assay^45^. The results were expressed as millimeters of inhibition zone diameter for the microbial growth surrounding the well of the investigated sample.

#### Antifungal activity

Composites were examined against the growth of several types of toxigenic fungal strains. This experiment evaluated their effectiveness in limiting fungal vegetative development and mycotoxin production. The emulsions of composites were evaluated in the fungal growth of liquid media using the previous methodology described by Khojah et al.^46^. The aqueous media of yeast extract sucrose (YES) containing *A. parasiticus* ITEM 11 spores, *Fusarium culmorum* KF191, or *Alternaria alternate* were administered separately using composite emulsions. The investigation was divided into treated and controlled groups of flasks for each fungal strain. After up to six days of incubation, the flasks were examined for fungal growth reduction (22 ± 2°C). They used the methods to quantify the inhibition ratio of the control flasks as a decrease in mycelia of fungal growth^47^.

### Application of selected emulsions against aflatoxins-producing fungi

Based on the antifungal activity recorded in the previous section (sec. 2.8), six composites were chosen to evaluate their activity against aflatoxigenic fungal strain activity of *Aspergillus flavus* in food packaging as a preservation using Fakhouri’s theory^48^. The base to select better composites out of their total (E1 – E16) was to evaluate the component’s interaction of composites and their impact on bioactivity as an anti-mycotic and anti-mycotoxigenic agent. These emulsions were distributed as follows:

E1: primary emulsion contained CMC and SH only.

E3: emulsion loaded with JOE and POE (free of shellac),

E4: emulsion loaded with JOE and POE (free of CMC),

E5: emulsion loaded with low jojoba (free of POE),

E10: emulsion loaded with high POE concentration (low JOE),

E14: The emulsion is loaded with high JOE and POE.

These emulsions were evaluated for their activity against toxigenic fungal growth and aflatoxins production in simulated media growth using yeast extract sucrose (YES) as liquid synthetic media and on corn grain as an application experiment.

#### Application in liquid growth media

Seven conical flasks (500 mL, in 2 sets), which were inoculated using *Aspergillus flavus* ITEM 698 suspension for a final concentration of 1.7 × 10^3^ spores/g, were utilized to evaluate the previous emulsions (E1, E3, E4, E5, E10, and E14), where the control flask (contains fungi spores only). Mycelia and aflatoxin reduction were evaluated as described before^49,50^. After five days of fungal growth in media (22 ± 2℃/5 days), it was filtered using filter paper (Whatman No.1), and then the paper was dried using a hot air oven until a three-constant weight. The reduction ratio was calculated as a percentage of control growth in the control fungal flask. However, the reduction of aflatoxins was determined in another set of flasks (12 days/ 28 ± 2 ℃).

#### Application in cereal preservation

The main idea was to compare the activity of composite substituents’ interaction with their impact on toxigenic fungal development and their products on stored grains. The previous emulsions were applied as liquor for cereal grain preservation against toxigenic fungi contamination. The evaluation process was performed using the selected composite materials to evaluate their capacity for aflatoxin limitation, according to Nazareth et al.^28^, with some modifications. Cereal grains (10 g; moisture content = was 13.2 ± 1.25%) were separated into 120 mm Petri dishes, placed on a rigid base, and transferred to 10 L jars. Samples were contaminated with *Aspergillus flavus* ITEM 698 suspension for a final concentration of 1.7 × 10^3^ spores/g. The jars were stored for 15 days at room temperature to allow the fungi to stabilize. Petri dishes containing 10 mL of saturated salt solution were also used to generate different relative humidity (RH) inside the jars. Jars were hermetically closed for 24 h to allow total volatilization and contact with the contaminated grains. Finally, jars were opened with small holes in the lids to allow ventilation. Total fungal colonies of *Aspergillus* were calculated using the same methodology described by Abu-Sree et al.^51^.

#### Determination of aflatoxins in simulated experiments

By the end of simulated experiments, the YES media of the set targeted aflatoxins evaluation was filtered using filter paper (Whatman No.1) and collected in a screw cap bottle for the assessment. Again, the set targeted to investigate the aflatoxins content in corn experiment was crushed, extracted using aqueous methanol^52^, and the filtrate was kept in screw cap bottles. Collected filtrates were investigated for their content of aflatoxins (AFB_1_, AFB_2_, AFG_1_, and AFG_2_). The quantitative analyses used a pre-calibrated fluorometer (VICAM Series 4EX Fluorometer, Watertown, MA, USA; limit of detection 1.0 ng/Kg). As per the approach outlined by Hafez et al.^53^, a 1 mL aliquot of the solution was combined with 10 mL of distilled water. Subsequently, this mixture was applied onto an Afla-test immunological affinity column and subjected to two rinses with 10 mL of distilled water at a 6 mL/min flow rate. A volume of 2 mL of methanol was used to elute the contents of AFs by applying it to the Afla-test column at a flow rate of 0.5 mL/min. The elution of the AFs content was then evaluated using a VICAM fluorometer.

### Molecular docking

The crystal structures of several enzymes associated with bactericidal and bacteriostatic effects, including isoleucyl-tRNA synthetase, DNA gyrase, dihydropteroate synthase, D-alanine: D-alanine ligase, IV topoisomerase, dihydrofolate reductase, and penicillin-binding protein 1a were obtained from the Protein DataBank website (accessed on August 17, 2022) using their respective PDB IDs: 1JZQ, 1KZN, 2VEG, 2ZDQ, 3RAE, 3SRW, and 3UDI. Meanwhile, the essential enzymes involved in aflatoxins biosynthesis, such as polyketide synthase (A0A1R3RGK0), non-ribosomal peptide synthase (B8N0E8), cytochrome P450 monooxygenase (A0A1R3RGJ7), and halogenase (A0A1R3RGJ2), were acquired from the UniProt database (accessed on February 3, 2022, and December 4, 2023). The enzymes and receptors were prepared by removing water and co-crystallized ligands and ions. The Pymol software version 2.5.1 was used to protonate them. Additionally, the 3D structures of the ligands were optimized using Avogadro Software version 1.2.0 and the MMFF94 force field. Finally, a web-based program called CB-DOCK2 was used for blind docking. The program was accessed via http://clab.labshare.cn/cb-dock/php/ on November 26–29 and December 2–5, 2023. The ligands were downloaded from the PubChem database and accessed through http://pubchem.ncbi.nlm.nih.gov/ on November 22–23, 2023. CB-DOCK2 uses OpenBabel and MGL Tools to convert input files to pdbqt format, predict protein cavities, and submit the top N cavities (n = 5 by default) to AutoDock Vina for docking. The final results are displayed after N rounds of computation. CB-Dock2 outperforms other blind-docking tools, with success rates for top-ranking poses whose RMSD was within 2 Å from the X-ray crystal structure position. The best-docked complexes are analyzed using Discovery Studio software (Ver. 21.1.0.20298)^54^.

### Statistical analysis

All tests were performed in triplicates, and the data were reported as means ± the standard deviation. Statistical software for the social sciences (SPSS V.16) was used to analyze the data. The analysis of variance (ANOVA) was used to assess the significant difference between the mean values, and Duncan’s multiple range test was performed (p = 0.05).

## Results and discussion

### Phenolic and flavonoid content of pomegranate peel extract

The phenolic fractions of pomegranate peel extract reflect its valuable content of phenolic and flavonoids (Table 2). The results referred to gallic, cinnamic, and ellagic acids as the higher phenolic acids determined in the POE, with ellagic content of 34.53 ± 2.54 mg/Kg. Furthermore, catechin, epicatechin, and rutin were recorded at 84.55 ± 3.37, 53.56 ± 2.24, and 47.24 ± 3.02 mg/Kg POE, and they are the most abundant flavonoids determined with valuable content in the POE. The content of Apigenin (39.66 ± 1.36 mg/Kg) and quercetin (36.41 ± 1.58 mg/Kg) are also defined in significant amounts for the POE.

**Table 2:** Fatty acid composition of Jojoba oil utilized in film material (calculated as a percentage).

	Jojoba oil fatty acid composition		
No.	Fatty acid	Symbol	FAC (%)
1	Oleic acid	C18:1	16.91 ± 0.54
2	Linoleic acid	C18:2	0.41 ± 0.005
3	Linolenic acid	C18:3	0.47 ± 0.03
4	Gondoic acid	C20:1	77.65 ± 1.74
5	Erucic acid	C22:1	4.14 ± 0.86
	Total	–	99.58 ± 0.64

Pomegranate peel extract content of phenolic and flavonoid compounds			
Phenolic acids	Amount (mg/Kg)	Flavonoid compounds	Amount (mg/Kg)
Gallic acid	95.47 ± 3.11	Catechin	84.55 ± 3.37
Ellagic acid	34.53 ± 2.54	Epicatechin	53.56 ± 2.24
Protocatechuic	7.55 ± 2.43	Catechol	0.55 ± 0.08
Ferulic acid	7.22 ± 1.56	Rutin	47.24 ± 3.02
Trans-ferulic acid	17.25 ± 1.34	Apigenin	39.66 ± 1.36
Cinnamic acid	41.16 ± 2.08	Quercetin	36.41 ± 1.58
Syringic acid	15.28 ± 2.51	Luteolin	19.54 ± 1.66
Caffeic acid	21.16 ± 2.33	Hisperdin	25.33 ± 2.81
Vanillic	27.14 ± 2.18	Naringenin	10.64 ± 1.67
*P-hydroxy* *Benzoic acid*	2.67 ± 0.41	Kaempferol	14.27 ± 1.05

Results were expressed as mean ± SD (SD: standard deviation).

FAC: fatty acid composition; phenolic compounds are expressed in milligrams per Kilogram (mg/Kg).

### Fatty acids composition of JOE

The JOE was utilized as a component of the formed bioactive composites in our present investigation. Regarding the results in Table 2, the fatty acid composition of the JO consists of monounsaturated fatty acids (98.70%) and polyunsaturated fatty acids minor content (0.88%), with an absence of saturated fatty acids content. It is worth mentioning that the significant long-chain fatty acid was gondoic (C 20:1 omega 9), followed by oleic acid (C 18:1; 16.92%) and erucic acid (C 22:1 4.24%). Omega 6 (C 18:2) counted 0.40%, while omega 3 (C 18:3) represented 0.49%.

Jojoba oil, a biologically active substance, is considered wax rather than triglyceride since it combines long-chain fatty esters (up to 98%). Unlike vegetable oils and animal fats, it consist of derivatives from fatty materials of acids or alcohols^55^. It mainly comprises omega-9 monounsaturated fatty acids, particularly 20:1 (11-eicosenoic acid, also called gondoic acid), 18:1, and 22:1 (erucic) fatty acids, which are connected to the 20:1, 22:1, and 24:1 fatty alcohols. Since each double-bonded alkyl component contains one, it has esters ranging from C38 to C44. The wax is highly oxidation-resistant since methylene has not broken double bonds^56^. Jojoba oil is a liquid wax used in cosmetics that can trap moisture and has many beneficial properties, such as analgesic, antipyretic, anti-inflammatory, antioxidant, antibacterial, and anti-parasitic activities. Mixing it with other substances stabilizes and retains its benefits for a long time^52^.

### Determination of total phenolic, total flavonoids, and antioxidant activities

Before their dryness, the emulsions were estimated to evaluate the expected activity of the formulated composite materials. The composites with higher total phenolic are ordered as E12, followed by E13, E14, E11, and E16. The emulsion formulas E4, E2, and E3 had the lowest phenolic content of the prepared composites (Table 3). Again, E15, followed by E12 and E11, is recorded as a higher composite in the flavonoid content; for E13 and E14, flavonoid content was considerable. Regarding antioxidant activity, the POE is estimated to be close to the standard reference of ascorbic acid but decreases in proportion to its amount in the composite emulsion.

**Table 3:** Total phenolic, total flavonoid, and antioxidant activities of composite emulsions.

	Total phenolic (mg GAE/100g)	Total flavonoids (mg QE/100g)	DPPH _(IC50)_ (mg TE/g)	ABTS^±^ _(IC50)_ (mg TE/g)
POE	147.84 ± 4.05^a^	69.32 ± 1.54^a^	9.81 ± 7.34^m, n, p^	10.74 ± 10.41^m, n, p^
JOE	12.72 ± 1.55 f.	6.31 ± 1.81 f.	229.51 ± 1.08^a^	235.77 ± 1.54^a^
E1	1.05 ± 0.36^g^	nd	83.25 ± 1.41^d^	83.08 ± 1.22^d^
E2	0.71 ± 0.27^g^	nd	97.34 ± 2.18^b^	94.29 ± 1.64^c^
E3	0.88 ± 0.11^g^	10.52 ± 0.48^e^	54.99 ± 2.27^h^	55.54 ± 1.81^h^
E4	0.28 ± 0.05^h^	11.08 ± 0.79^d, e^	88.52 ± 1.34^c^	101.51 ± 2.71^b^
E5	1.23 ± 0.88^g^	nd	77.27 ± 1.94^e^	79.18 ± 1.05^e^
E6	5.89 ± 0.67	0.54 ± 0.05^h^	74.01 ± 2.05 f.	73.97 ± 1.02 f.
E7	16.55 ± 2.71 f.	1.18 ± 0.08^g^	64.08 ± 2.31^g^	64.02 ± 1.37^g^
E8	27.38 ± 1.54^e^	2.08 ± 0.09 f.	48.94 ± 1.88^i^	45.49 ± 1.34^i^
E9	41.28 ± 2.18^c^	9.41 ± 0.74^e^	19.66 ± 1.47^k^	17.94 ± 1.47
E10	41.28 ± 2.16^c^	11.11 ± 1.56^d, e^	19.21 ± 1.74^k^	19.28 ± 1.29^k^
E11	42.16 ± 1.88 ^c, d^	13.86 ± 1.18 ^c^	13.31 ± 1.66^m, n^	13.22 ± 2.08^m, n^
E12	47.3 ± 2.08^b^	14.02 ± 1.69^c^	11.66 ± 1.89^n^	12.61 ± 1.66^n^
E13	44.08 ± 2.64^b,c^	12.96 ± 1.05^c, d^	13.66 ± 2.08^m^	13.15 ± 1.54^m^
E14	44.69 ± 2.06^b,c^	12.95 ± 1.37^c, d^	13.12 ± 1.64^m^	14.88 ± 1.27^m^
E15	40.66 ± 1.87^d^	17.08 ± 0.55^b^	21.28 ± 1.54^j^	21.21 ± 1.32^j^
E16	42.22 ± 1.02^c, d^	13.54 ± 0.36^c^	15.54 ± 1.84^m^	16.08 ± 1.78^m^
Ascorbic	–	–	7.71 ± 1.66^p^	8.08 ± 1.39^p^

Results were expressed as mean ± SD (n = 3;* P* = 0.5), TE: Trolox equivalent.

nd: not detected; Data represented by different superscript letter are significant for each column.

The correlation coefficient between total phenolic content and antioxidant activity was studied before^57^, and it was reported with R-value close to 0.93. Similarly, the correlation coefficient between total flavonoid content and antioxidant activity was calculated for the plant extract^57^; the R-value was recorded as equal to 1, reflecting the high Correlation. Also, recent studies pointed out the relationship between antioxidant activity and antimicrobial activity, which was indirectly joined with the content of phenolic compounds^58^. Flavonoids are potent scavengers of most oxidizing chemicals, including singlet oxygen and other free radicals implicated in various illnesses^59^.

The EC_50_ values of the radical scavenging activity of various soluble fractions of the extract and the concentrations of phenolics or flavonoids demonstrated a significant association, suggesting that phenolic acids and flavonoids may be the key contributors to antioxidant activity^60^. However, a non-significant connection was discovered in the case of hydrogen peroxide radical scavenging. Varied phenolic compounds have different reactions in the Folin-Ciocalteu technique. Similarly, depending on their chemical composition, the molecular antioxidant response of phenolic compounds varies dramatically^60^.

### Mechanical and physical properties of the dried composites

The mechanical properties of the prepared dried composites showed a variation in their properties. Three groups of film composites were evaluated after the film preparation: the first group represented different POE concentrations (E5-E9). The second group of composite films was represented by films E10, E11, E12, E13, and E14), which were prepared using several concentrations of Jojoba oil. The third film composite group is represented by E1, E2, E3, E4, E15, and E16 and is prepared to have CMC/shellac concentrations**.** The air permeability (AP) and mechanical strength are summarized in Table (4).

It was noticed that using different concentrations of POE (E5-E9, and E1), air permeability was increased until 7.5 mg of POE in E8 and 5 mg in E7 by 37.5% and 12.5%, respectively, than E1 (blank). In the second composite film type using different concentrations of jojoba, it was observed that increasing JOE until 7 mL, as in E13, shows an increase in AP by 500%. In comparison, it increases by 293.75% in E10 (1 mL JOE) than E1 (blank), respectively. However, in the third group of composite film, using different concentrations of CMC/shellac (E15-E16), it was clear that increasing CMC until 1.05 g in E16 shows a decrease in AP than E1 (blank). The best improvement in AP is in E13, then E10, E8, and E7 by 500%, 293.75%, 37.5%, and 12.5%, respectively; it is clear that air permeability is excellent for all concentrations of CMC due to comprehensive interference with shellac fibers in the presence of jojoba then POE as shown in Table 4.

**Table 4:** Mechanical properties of different composites.

Emulsion Film	Burst (Kg/cm^2^)	AP (Sec./100mL air)	TK (mm)	TS (MPa)	EB (%)	Stiffness (N/m)	Stress at Maximum Load (MPa)	Young’s Modulus (MPa)	Appearance
E1	0.55	160	0.48	7.69	24.6	294.55	0.77	1.27	Indicator
E3	1.05	170	0.55	6.97	30.6	277.15	0.69	0.89	Indicator
E5	0.75	120	0.63	5.41	21.2	302.66	0.54	0.92	Light-oil release
E6	2.1	85	0.65	4.43	18.9	321.75	0.44	0.79	Light-oil release
E7	1.45	180	0.55	7.86	20.6	356.18	0.78	0.55	Light-oil release
E8	1.17	220	0.45	0.48	20.3	377.37	0.48	1.02	Light-oil release
E9	0.85	160	0.48	5.82	18.4	381.59	0.51	0.99	Light-oil release
E10	1.41	630	0.32	2.97	30.8	394.19	0.27	2.12	excellent
E11	1.55	17	0.43	4.96	22.5	512.05	0.49	0.96	Light-oil release
E12	1.4	55	0.6	8.74	21.1	361.74	0.87	1.19	oil release
E13	0.95	960	0.73	8.59	19.6	374.15	0.85	0.5	oil release
E14	0.35	nil	0.66	2.97	20.6	399.86	0.29	1.03	acceptable
E15	1.15	110	0.51	4.66	27.8	455.42	0.4	0.59	oil release
E16	0.6	74	0.55	8.84	34.5	1237.93	0.88	1.57	acceptable

AP: Air permeability 100 mL air (sec), TK: thickness, TS: Tensile strength, EB: Elongation at the break;

+ F2 and F4 composites did not form a suitable film for the measurements (crushed).

Burst strength for the first composite film group (E5-E9) shows an excellent improvement of burst character by adding different concentrations of POE, as shown in Table 4. The order of improvement was E6; 281% (POE is 2.5%), more than 163% in E7, and 112% in E8, compared with blank E1. Also, the second composite film group exhibits a good increase in burst properties with the addition of Jojoba (E10-E14), as shown in composite E11; 181% (jojoba oil is 3%) then 156% in E10 and 154% in E12 compared with the blank E1. While the third composite film group, which is prepared using different concentrations of CMC/shellac (E15-E16) and a constant percent of jojoba oil and POE, shows a moderate burst character as shown in composite (E15; 109% then 9% in E16) when compared with the blank E1, Table 4.

From the above, it was achieved that the best burst strength was obtained with the addition of 0.075 mg POE (E6, 2.1 kg/cm^2^, 281%, class 1), then E11 (181%, 3 mL jojoba oil, class 2). At the same time, composites (E1& E16) show the lowest burst values, 0.55 and 0.6, respectively. Composites’ burst and air permeability were excellent due to the most compatible ratio between CMC and shellac. Strength properties, including tensile strength, elongation at break, stiffness, and Young’s Modulus, were determined to illustrate the effect of various constitutions of the different composites. The obtained results are shown in (Table 4). Tensile strength and Deformability (Young’s Modulus) clearly show that the first type, increasing POE until 0.15 mg in E7, increases tensile strength by 2% more than E1 (blank). Our findings agreed with those obtained before Kumar; the films’ tensile strength (32.45–35.23 MPa) rose as the volume percentage of pomegranate peel extract increased^61^. Also, the films’ tensile strength (1.83–2.91 MPa) rose after adding active material (pomegranate peel extract % increased) to the test, which agreed with the previous investigation results^62^.

Also, in the second type, increasing JOE until 5 and 7 mL, as in E12 and E13, shows an increase of 12% and 10.5% in tensile strength than E1 (blank), respectively. In addition, the third type (E15-E16) increased in CMC until 1.05 g in E16, showing an increase of 13% in tensile strength compared to E1 (blank). Generally, the best improvement in tensile strength is in E16, which has the highest CMC content then E12, which contains 5 mL JOE; E13, which includes 7 mL JOE; finally, E7, which contains 0.15 mg POE, by 13%, 12%, 10%, 2% respectively, as in Table 4.

Regarding the results representing the elongation at break (EB), the best improvement in Elongation was E16, followed by E10 and E15 by 40%, 25%, and 13%, respectively. Our results aligned with those of Galus, S. et al., who found that elongation improved noticeably when jojoba oil content increased (from 48.4 to 101.1%, for the highest percentage)^63^. Nevertheless, for all samples of the first group (E5-E9), it decreased more than the E1 (blank), as in Table (4). A study of deformability (Young^’^s Modulus) is essential for the shape retention of natural composites. The deformability depends, to a large extent, on its structure as well as its stability. It is determined by the modulus values in Table 4, which clearly show that composite E13 is the best, with lower deformability, followed by E7 and E15, compared with other composites. However, composite E10 (1 mL JOE) showed a 66% increase in Young^’^s Modulus in the second group (CF2), followed by E16 (23% increase) in the third type of composites compared with the blank composite E1. Consequently, more significant stress is required for its deformation. The observed reduction in deformability can be explained due to the stability of the composite^64^.

The obtained results of stiffness and stress illustrate the efficacy of the constitution of the prepared natural composites. Where Stiffness character in this connection, it was observed from Table (4) that when using different concentrations of POE (E5-E9), the stiffness increase with increasing in POE, the best stiffness was exhibited with composite in ordered E9, E8, then E7 by 29%, 28% and 20% respectively, more than the blank E1; and using different concentrations of Jojoba (E10-E14, CF2) show high Stiffness character. The best stiffness was exhibited with composite in ordered E11, E14, and then E10 by 73%, 35%, and 33%, respectively, when compared with the blank E1. The third group, using different concentrations of CMC/shellac (E15-E16), shows good Stiffness character, exhibiting an excellent increase as shown in composite E16; 320% then 54% in E15 when compared with the blank E1 as in Table 4. The stress properties, E16 exhibits a high-stress value because it contains a high percentage of CMC/ shellac (1.05:0.15) loaded with ratio 3:0.3, mL/mg of JOE/ POE, then E12 (JOE/ POE, 5:0.3) and finally, E7 (JOE/ POE, 3:0.225) by 14.28%, 12.99%, 1.30% respectively as in Table 4.

Generally, the results obtained in Table (4) illustrate the efficacy of the constitution of the prepared natural composite. The enhancements were observed in tensile strength elongation at break and burst properties of the CMC/ shellac composite, which is flexible and more elastic. These outcomes were consistent with those of Mohamed et al., who detected a significant improvement in the mechanical characteristics of CMC with the increase in shellac content^34^. This may be due to the formation of the chemical bond between shellac carboxylate groups with hydroxyl groups of CMC and glycerol in the presence of JOE/ POE. Also, the presence of shellac between CMC fibrils reduces the hydrogen bonding between CMC layers due to more spacing between the fibrils^34^. Also, the improvement of properties may be referred to as the electrostatic forces between shellac’s side chain function groups with CMC, which acts as a good cross-linker. It is worth mentioning that the solubility defect of shellac was improved by all proportions added to CMC. Table 5 compares the composites’ main functional properties of the previous and present study.

**Table 5:** Compared the main functional properties of the composites in previous and current studies.

Composite’s components	Primary findings	References
*Pomegranate peel extract was added to chitosan-based edible films at varying doses (0.2–1.0 g/mL)*	The films’ tensile strength (32.45–35.23 MPa), thickness (0.142–0.159 mm), phenolic content (5.75–32.41 mg/g), and antioxidant activity (23.13–76.54%) increased as the volume percentage of pomegranate peel extract increased	^60^
*Pomegranate peel extract was added to chitosan films in the presence of glycerol and choline chloride, employed as the hydrogen bond donor and acceptor, respectively*	The increase in pomegranate peel extract percentage rise in the films’ tensile strength (1.83–2.91 MPa)	^61^
*Jojoba oil addition on hydrogel edible films based on whey protein isolate*	The elongation significantly improved when the jojoba oil content was raised (from 48.4 to 101.1%, for the highest percentage)	^62^
*Innovative natural composite films using shellac, gelatin, and/or carboxymethyl cellulose (CMC)*	The films are flexible, without aggregation, and have good antibacterial and tensile strength, homogeneity, transparency, and elongation properties. More CMC improved the breaking length of composite films and the elongation at break. CMC/shellac films exhibit significantly reduced tensile strength and increased air permeability and have greater flexibility than pure CMC film	^33^
*carboxymethyl cellulose/shellac (CMC/SH), and loaded with pomegranate extract (POE) with or without jojoba oil (JOE) at various concentrations*	An enhancement was recorded for tensile strength and elongation at break and burst properties of the composites, where the results point out the amelioration of flexibility and elasticity. Air permeability was increased up to 37.5, 500, and 500%; Burst strength was increased up to 281, 180, and 109%; Tensile strength was increased up to 2, 12, and 13%; The elongation at break was increased up to -ve, 40, and 25%; Young’s Modulus was increased up to -ve, 66, and 23%; Stiffness was increased up to 29, 73, and 320%; The stress properties was increased up to 1.30, 12.99, and 14.28% for POE, JOE, and CMC/shellac, respectively	The present study

### FT-IR analysis

**Fig. 1:**
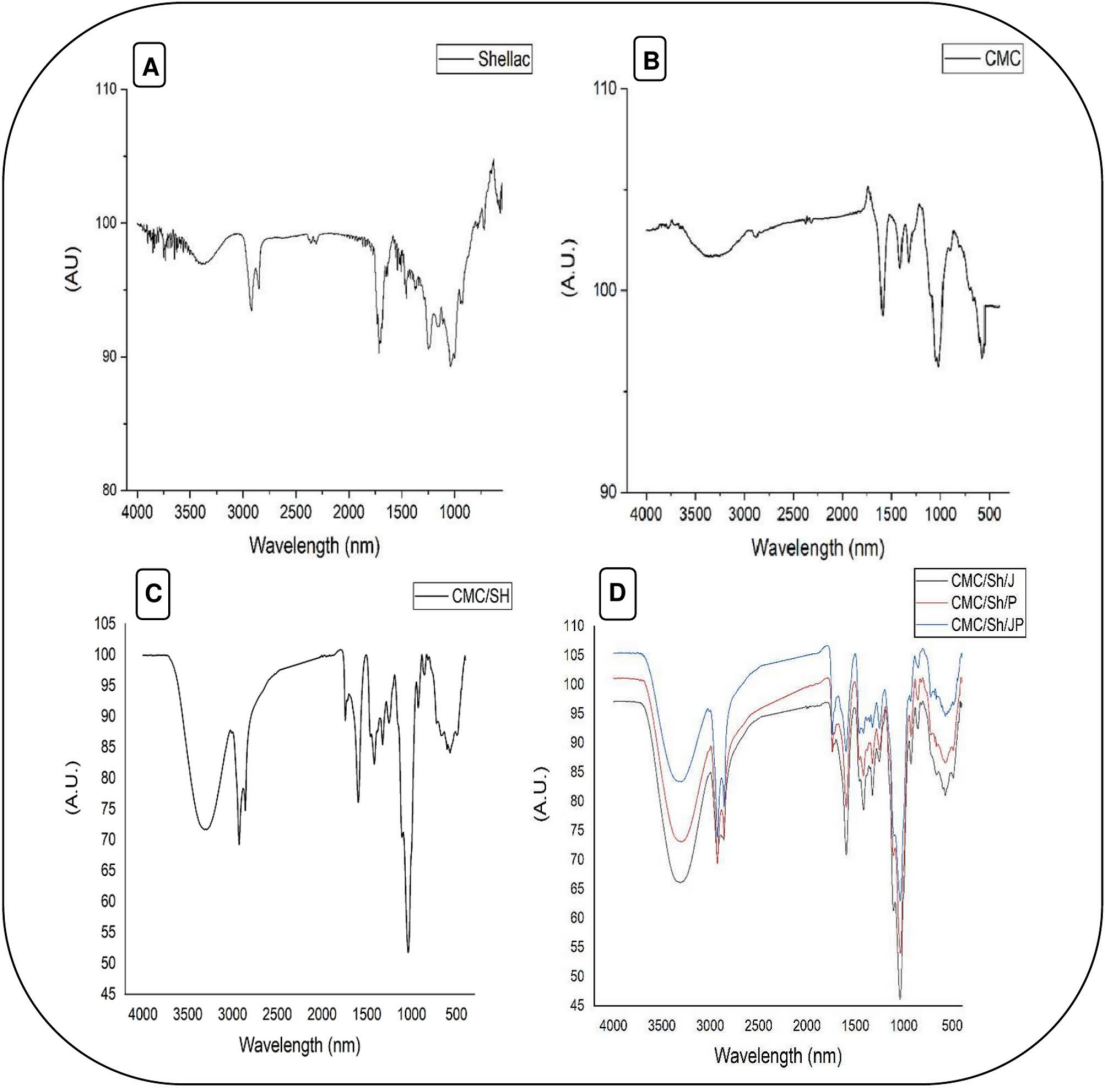
FT-IR spectra of Shellac (**A**), CMC (**B**), Shellac/CMC (**C**), and Shellac/CMC/POE/JOE (**D**). CMC: carboxy-methyl cellulose; Sh: shellac; J: jojoba; P: pomegranate; CMC/Sh/J: a composite of carboxymethyl cellulose and shellac loaded by jojoba oil; CMC/Sh/P: a composite of carboxymethyl cellulose and shellac loaded by pomegranate extract; CMC/Sh/JP: a composite of carboxymethyl cellulose and shellac loaded by jojoba oil and pomegranate extract.

FT-IR is one of the most valuable techniques for material characterization. Figure 1 shows the ATR FTIR spectra of CMC, shellac, CMC/shellac, CMC/shellac/JOE, CMC/shellac/POE, and CMC/shellac/JOE/POE sample materials. The ATR FTIR spectra of CMC (Fig. 1A) revealed characteristic absorption bands at 2878 nm corresponding to C-H stretching. The intense peak at 1588 nm is related to the presence of COO^–^ -groups. The bands between 1411 and 1318 nm correspond to stretching in the plane and C-H stretching in the symmetry of CMC^65^. For purified shellac (Fig. 1B), the FTIR spectrum showed an O–H stretching peak at 3392 nm and two C–H stretching bands at 2921 and 2853 nm. The C = O stretching signal of the terminal -COOH group was observed at 1720 nm, accompanied by COO^-^ asymmetric and symmetric stretching around 1639 and 1458 nm, respectively. Additionally, stretching double bond vibration appeared at 1652 nm, and ester groups appeared as C–O–C asymmetric aliphatic ester stretches at 1251 nm^66^.

The spectra of the composites with extracts displayed a similar trend to those with free extracts or polymers used (Fig. 1C and D). However, peaks appearing in pure extracts or polymers were shifted to a higher or lower frequency in the composite. Also, the intensity of the peaks in the composite was reduced compared to peaks of pure extracts or polymers, indicating the interaction between the composite components^67^. For example, the aliphatic O–H stretching shifted to a lower region at 3294 nm. At the same time, C-H, carbonyl, COO-, and C–O–C asymmetric and symmetric stretching showed a higher shifting to 2925, 1732, 1592, 1412, and 1322 nm. The frequencies at 923 and 852 nm are related to the flavonoid rings. The asymmetric C–O–C aliphatic ester stretching has the same frequency as shellac at 1251 nm (Fig. 1D).

Noteworthy, the FT-IR spectra of the JOE based on its fatty acids composition are supposed to have carbonyl double bonds (C = O stretching) of the free fatty acids, = C–H stretching vibration, C–H stretching vibrations, and methylene (–CH2–) and methyl (–CH3) stretching vibrations^68^. All previous peak frequencies in the composite spectrum included the extracts, leading to the above-shifting. According to Hady et al.^69^, the significant peaks obtained for POE were at 3321 cm^*-*1^ peak corresponded to -OH groups, 2968 cm^*-*1^ refers to the asymmetric stretch of the C-H in methyl group, which is present in most phenolic compounds, while 878 cm^−1^ is probably related to the aromatic ring of flavonoids. Again, all of the previous frequencies were found in the composite spectrum obtained in the current study but at different shifts due to the interaction between the extract components and the polymers.

### Antibacterial activity of composite

The antibacterial evaluation of emulsion composites against four strains of pathogenic bacteria is shown by the efficiency of E16, which has the highest antibacterial activity (Table 6). The emulsion composite of E2 is recorded with the lowest antibacterial activity. Compared to Azithromycin’s standard antibiotic (ST), antibacterial activity is positively related to CMC and POE contents in the emulsion composites. It was noticed that E10 could be considered an emulsion with mediating anti-pathogenic activity. Moreover, antibacterial inhibition is recorded as meaningful for the crude application of the POE. The efficiency of jojoba oil against applied strains of bacterial pathogens is limited, and the recorded zone of inhibition was minimal.

**Table 6:** Antibacterial effect of composite emulsions determined against pathogenic strains.

	*Gram-positive strains*		*Gram-negative strains*	
	*Staphylococcus aureus*	*Bacillus cereus*	*Salmonella typhi*	*Pseudomonas aeruginosa*
ST	34.41 ± 0.21	34.37 ± 0.34	31.18 ± 0.44	32.31 ± 0.51
POE	24.15 ± 1.27	25.05 ± 1.51	26.33 ± 1.79	26.08 ± 2.02
JOE	2.66 ± 1.14	2.18 ± 1.33	2.18 ± 1.02	3.41 ± 1.34
E1	4.67 ± 0.24	4.97 ± 0.05	5.58 ± 0.02	4.41 ± 0.01
E2	0.37 ± 0.03	1.34 ± 0.37	0.67 ± 0.04	0.75 ± 0.04
E3	10.23 ± 0.41	11.97 ± 0.81	11.15 ± 0.61	11.59 ± 0.61
E4	7.41 ± 0.47	7.82 ± 0.96	6.37 ± 0.84	6.08 ± 0.73
E5	5.27 ± 1.25	6.67 ± 0.87	5.73 ± 1.05	5.64 ± 0.96
E6	9.34 ± 0.39	8.71 ± 1.37	9.55 ± 1.03	9.28 ± 1.36
E7	13.43 ± 1.76	13.51 ± 1.22	14.46 ± 1.43	11.81 ± 1.37
E8	15.33 ± 0.88	15.74 ± 1.34	14.72 ± 0.21	14.07 ± 1.38
E9	9.24 ± 0.33	9.54 ± 0.05	8.33 ± 0.18	10.14 ± 0.97
E10	12.58 ± 0.17	11.88 ± 0.23	12.68 ± 0.21	11.94 ± 1.05
E11	12.14 ± 0.34	11.34 ± 0.54	11.37 ± 0.21	11.84 ± 0.69
E12	9.67 ± 0.33	9.34 ± 0.67	9.46 ± 0.34	10.24 ± 1.21
E13	8.34 ± 0.33	8.08 ± 1.02	8.67 ± 0.24	8.37 ± 0.92
E14	8.84 ± 1.34	8.67 ± 0.08	8.54 ± 0.41	9.08 ± 1.17
E15	11.09 ± 0.37	11.94 ± 0.56	12.05 ± 0.72	11.84 ± 0.69
E16	19.34 ± 0.05	18.66 ± 0.31	17.41 ± 0.08	18.55 ± 0.02

Results were expressed as mean ± SD (n = 3;* P* = 0.5).

ST: standard antibiotic of Azithromycin

Several investigations referred to the anti-pathogenic effect of the POE, partially against *Salmonella, Pseudomonas, Bacillus*^70^, and *Cronobacter*^71^. Moreover, it was reported as efficient in inhibiting the contamination connected to *E. coli* bacterial infection^72^. Furthermore, applying POE in composite film ameliorates antibacterial activity against Gram-positive and Gram-negative pathogenic bacterial strains^73^. This effect is connected to its valuable antioxidant content. The impact of POE on pathogenic bacteria was linked directly to the bioactive components such as punicalagin and pro-anthocyanins, which possess significant activity in inhibiting the growth of examined bacterial strains on the synthetic media^72^. Adding the POE to a hydrogel composite acts dual functions as antimicrobial (*against Escherichia coli and Staphylococcus aureus* ) and antioxidant potency that aids the shelf life extension of food material^74^

### Antifungal activity of composite

The impact of composite emulsions, the POE, and JOE was estimated on solid media against toxigenic fungal strains (Table 7). The results reflected the efficiency of several composite emulsions, besides the POE, in inhibiting the growth of the fungal strain with transparent zones. The effect of E10, E9, and E11 emulsions was recorded as having a higher inhibition effect than other composite emulsions. The inhibition zone recorded for E10 was close to the standard antifungal results and more efficient than applying crude POE alone against examined toxigenic fungal strains. Meanwhile, the inhibition achieved by the composite emulsions of E9 and E11 was recorded more or less as the POE effect against toxigenic fungal strains of the present experiment.

**Table 7:** Anti-mycotic effect of composite emulsions determined against mycotoxigenic fungal strains.

	*A. flavus*	*F. graminearum*	*P. verrucosum*	*A. niger*
ST	26.37 ± 0.41	31.88 ± 0.89	28.46 ± 0.67	29.74 ± 1.02
POE	18.15 ± 1.66	18.22 ± 1.47	18.71 ± 2.29	19.05 ± 2.18
JOE	8.17 ± 1.02	8.44 ± 1.77	8.69 ± 2.34	9.57 ± 1.54
E1	4.67 ± 0.86	5.47 ± 0.55	5.52 ± 1.14	5.35 ± 1.47
E2	3.39 ± 0.47	4.77 ± 1.14	4.76 ± 0.59	3.51 ± 1.08
E3	11.85 ± 2.11	11.91 ± 2.05	11.66 ± 1.81	11.08 ± 1.84
E4	9.41 ± 2.08	8.02 ± 1.41	8.91 ± 1.67	8.06 ± 1.55
E5	7.47 ± 4.11	6.97 ± 1.41	7.67 ± 1.54	6.87 ± 2.57
E6	9.39 ± 1.54	8.81 ± 1.37	9.33 ± 2.07	9.41 ± 1.15
E7	10.04 ± 1.05	10.73 ± 1.24	9.77 ± 2.34	10.51 ± 1.39
E8	13.17 ± 2.05	12.88 ± 2.18	13.55 ± 1.67	13.42 ± 2.11
E9	17.14 ± 1.54	18.23 ± 1.79	18.11 ± 1.34	18.41 ± 1.81
E10	21.75 ± 1.73	20.92 ± 1.15	21.25 ± 1.81	22.18 ± 1.08
E11	17.14 ± 1.21	17.94 ± 1.34	17.86 ± 1.14	18.11 ± 1.53
E12	12.34 ± 1.29	11.34 ± 1.17	11.67 ± 0.44	12.67 ± 1.37
E13	12.37 ± 1.15	14.55 ± 1.14	11.74 ± 1.54	12.47 ± 2.64
E14	13.18 ± 1.52	13.37 ± 1.14	13.35 ± 1.21	14.67 ± 1.55
E15	10.01 ± 1.11	11.17 ± 2.04	9.54 ± 1.31	10.37 ± 1.79
E16	12.45 ± 1.21	12.97 ± 1.44	11.31 ± 1.88	12.89 ± 1.73

Results were expressed as mean ± SD (n = 3;* P* = 0.5).

*flavus:Aspergillus flavus; F. graminearum:Fusarium graminearum; P. verrucosum: Penicillium verrucosum; A. niger: Aspergillus niger*

ST: standard antifungal of nystatin; POE: pomegranate extract; JOE: jojoba oil extract.

In general, pomegranate byproduct extracts were known to have an antifungal effect, such as that was reported against dermatophytes^75^ and *candida sp*^76^. The POE effect was also reported against fungal pathogens of horticulture crops like tomatoes^77^. The impact of peel extract was evaluated against the green mold of citrus infection, where the results point out the positive inhibition of *Penicillium* strains^78^. Another investigation evaluated the antifungal activity of plant extracts against *Fusarium* fungi, including the POE^79^. The data declared the POE’s significant effect on inhibiting fungi’s mycelia growth. The study also mentions the Correlation between the phenolic compounds and their antifungal activity, which was reported at R = 0.69^79^. Recent investigations illustrated the anti-mycotoxigenic effect of the extract against aflatoxigenic and mycotoxigenic fungi^80,81^.

The effect of pomegranate extract may be extended to reduce the fungal secondary metabolite secretion^81^. Because the pomegranate byproduct contributes to more than half of the fruit’s weight, it is a significant source of bioactive components. Punicalagin and gallic acids, in particular, are the principal active components in the peel and have been linked to the extract’s antibacterial properties^46,82^. Furthermore, because POE combines direct antifungal efficacy with toxin production suppression, they can be employed against mycotoxigenic fungi^83^. A methanolic extract of pomegranate byproduct considerably inhibited the conidial germination and hyphal elongation rate of *Aspergillus* and *Fusarium*^84^. Also, by combining pomegranate byproduct extract with azole fungicide, the aflatoxin creation was decreased by 97%, and their genetic pathway was blocked^76^.

### Determination of the emulsions efficiency for anti-aflatoxigenic fungal inhibition

A simulated media was utilized to evaluate the composite efficiency to inhibit the growth of high aflatoxin-producing strain. In light of preliminary laboratory trials, six types of composites were chosen for the evaluation experiment in two simulated media (liquid growth media, Corn seed coating application), which are contaminated by the fungal spore suspension. The result reflected the gradient efficiencies of emulsion compositions in the mycelial weight reductions (Table 8). It was noticed that E10 was the most efficient emulsion that achieved more mycelial inhibition. The emulsion with 10mg of the POE showed an immediate inhibition effect, even if the oil content was changed. This point can be joined to the recorded oil release to separate from the composite emulsion, and it could be referred to as the more oil quantity that is not distributed in the composition structure.

**Table 8:** Composite formulas influence reducing *Aspergillus* mycelia weight and its related aflatoxins (in liquid media and on corn).

*liquid media simulation of fungal infection and aflatoxins*							
	Mycelia weight	Mycelia inhibition (%)	AFB_1_ (ng/g)	AFB_2_ (ng/g)	AFG_1_ (ng/g)	AFG_2_ (ng/g)	Total Afs (ng/g)
Cont	12.5741 ± 1.56	–	365.41 ± 5.22	181.63 ± 2.81	211.51 ± 3.44	127.34 ± 1.69	885.89 ± 13.16
E1	7.6188 ± 1.05	39.41%	289.13 ± 2.81	149.66 ± 2.14	161.37 ± 3.05	101.97 ± 1.21	702.13 ± 9.21
E3	8.9692 ± 1.67	28.67%	291.56 ± 2.47	154.08 ± 3.51	163.58 ± 2.74	104.51 ± 2.08	714.55 ± 4.24
E4	10.2861 ± 1.41	18.19%	306.88 ± 3.21	177.96 ± 3.14	196.41 ± 5.22	116.24 ± 2.74	702.13 ± 5.77
E5	5.5446 ± 0.96	55.91%	116.21 ± 1.08	121.25 ± 1.37	122.64 ± 2.18	96.57 ± 1.08	456.67 ± 5.71
E10	1.9979 ± 0.54	84.11%	11.36 ± 2.54	nd	5.41 ± 1.81	nd	16.77 ± 4.35
E14	4.2774 ± 0.88	73.94%	44.51 ± 1.02	19.66 ± 1.21	26.72 ± 1.02	9.87 ± 0.88	100.76 ± 4.13

*Corn seed simulation of fungal infection and aflatoxins production*							
	Mycelia weight	Mycelia inhibition (%)	AFB_1_ (ng/g)	AFB_2_ (ng/g)	AFG_1_ (ng/g)	AFG_2_ (ng/g)	Total Afs (ng/g)
Cont	11.2826 ± 2.37	–	288.53 ± 7.16	137.12 ± 3.24	144.26 ± 4.05	115.66 ± 3.14	685.57 ± 17.59
E1	7.6188 ± 0.81	32.47%	268.34 ± 2.21	125.88 ± 1.26	134.45 ± 2.08	106.75 ± 2.76	635.42 ± 8.31
E3	6.4641 ± 0.37	42.71%	211.94 ± 1.89	97.92 ± 3.08	103.20 ± 3.66	79.72 ± 2.18	492.78 ± 10.81
E4	8.9894 ± 0.24	20.33%	253.91 ± 3.05	122.04 ± 2.81	128.97 ± 3.47	103.16 ± 2.34	608.08 ± 11.67
E5	5.2851 ± 0.31	53.17%	106.95 ± 2.97	62.25 ± 2.37	52.81 ± 2.84	39.11 ± 3.17	261.12 ± 11.35
E10	1.6952 ± 0.18	86.52%	nd	nd	nd	nd	nd
E14	2.1718 ± 0.26	82.73%	nd	nd	nd	nd	nd

Results were expressed as mean ± SD (n = 3;* P* = 0.5). nd: not detected; Cont.: the control values without the treatment application in media.

The oil content from jojoba in the emulsion composite of E10 was 1mL oil per 60 mL of emulsion composite (1.66%). This ratio was fair enough to achieve the most inhibition impact of *Aspergillus flavus* growth in the liquid media. This strain is known for its ability to produce aflatoxins. As the application of emulsion composite reduced the mycelia growth, this pointed to the reduction of the fungi metabolites (primary or secondary metabolites). Metabolite reduction can be connected to mycelia weight reduction and biochemical changes that might happen due to the composite emulsion’s bioactive components.

### Evaluation of composite for the aflatoxins reduction

Implementing composite loaded with bioactive constituents into fungal media reflects a valuable impact, particularly for E10 composite, on reducing secretion levels of aflatoxins. The reduction impact of aflatoxins concentrations on corn seed coated by the composite loaded materials is recorded as significantly high for E10, followed by E14. It was noticed that applying composite coating on corn before the inoculation using fungal spores leads to a significant reduction in aflatoxins secretion on corn, similar to the results regarding liquid media used for the spore inoculation.

The result also reflects the absence of aflatoxins (AFB_1_, AFB_2_, AFG_1_, and AFG_2_) by applying composite coating on inoculated corn E10 and E14 treatments. These results could be joined to the composite content, concentrations of bioactive constituents loaded, and the composite structure. The results indicated a significant reduction in mycelial growth and aflatoxin production in corn seeds treated with composite coatings E10 and E14 containing jojoba oil and pomegranate extract.

Jojoba Oil is a natural wax ester that is recognized for its antimicrobial properties and ability to form a protective barrier^85^. Its hydrophobic nature deters moisture and fungal germination. Moreover, Pomegranate Extract is a rich source of polyphenols and antioxidants and has antifungal and antibacterial properties that can inhibit microbial growth of pathogens and toxigenic fungi^86^. The difference in jojoba oil content between E10 (1 mL) and E14 (10 mL) may impact the effectiveness of the treatment. Higher concentrations enhance the antifungal activity and protective barrier. Still, it was recorded to have a high oil release during the film dryness process (which recommends its application for drying directly on targeted materials). Again, it is essential to point out that the efficiency of the coating application can affect adhesion and uniformity, potentially influencing the effectiveness of the barrier against fungal invasion.

On the other hand, the coating alters the microenvironment around the seed, impacting moisture retention and gas exchange and making it favorable for a more hostile environment for fungal growth^87^. Significantly, both jojoba oil and pomegranate contents can create a physical barrier, inhibiting spore penetration and moisture accumulation. This leads to reduced conditions conducive to fungal germination and growth.

The application of composite coatings E10 and E14 containing jojoba oil and pomegranate extract effectively reduced both mycelial growth and aflatoxin production in corn seeds. The combination of physical barrier formation, antimicrobial properties of the constituents, and environmental modifications led to a significant decrease in fungal activity and aflatoxin levels.

Jojoba Oil can act to disrupt the cytoplasmic membrane of fungi, leading to cell lysis and ultimately inhibiting mycelial growth^88^. Its hydrophobic properties may create an unfavorable environment for fungal spores. Moreover, phenolic compounds of pomegranate extract inhibit enzyme activity essential for fungal metabolism, reducing growth rates and aflatoxin production potential^86^. Also, the coatings’ components may bind to crucial nutrients that the fungi require for growth, limiting their availability and impacting fungi proliferation. The application of these coatings may stimulate a defense response in the corn seeds, potentially leading to the production of secondary metabolites that could inhibit fungal development.

Several factors are suggested to lead to the aflatoxin reduction occurrence, such as: 1-direct inhibition of mycelial growth, where mycelial inhibition is the most significant factor is the drastic reduction in mycelial weight (86.52% for E10 and 82.73% for E14), and directly correlates with the observed non-detection of aflatoxins (nd). Without substantial fungal growth, the potential for aflatoxin production is drastically lowered; 2 – The composite coatings likely modify the local environment around the seeds, minimizing favorable conditions for fungi, such as moisture retention and nutrient availability, which are essential for fungal growth. 3 – The combination of jojoba oil and pomegranate extract may produce synergistic effects that enhance antifungal activity beyond what is achieved by either component alone. 4 – The coatings’ protective qualities not only reduce fungal growth but could also safeguard the seeds from spoilage, contributing to longer shelf life and better viability.

### Determination of the best emulsion characteristics

**Fig. 2:**
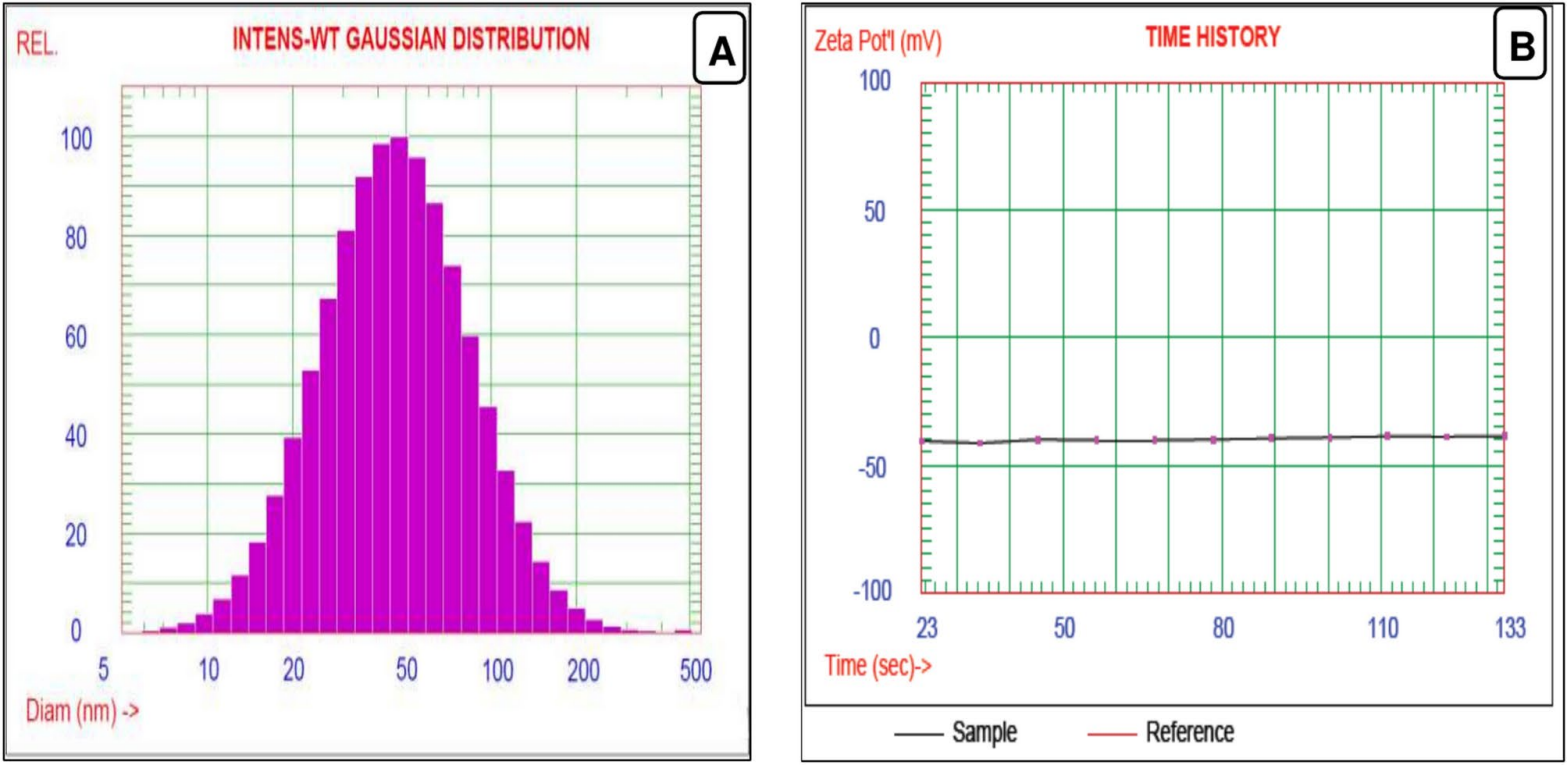
(**A**) particle size and (**B**) zeta potential determined for the chosen best emulsion formulation of E10.

The emulsion of E10, which represents the best results for the antimicrobial, antifungal, and anti-aflatoxigenic properties, was chosen to evaluate its characteristics. The formed emulsion showed Fig. 2 a unique particle size value (54.81 ± 1.46 nm), ζ- potential (-38.74 ± 0.22 mV), and a considerable value of the polydispersity index (31.12 ± 1.02). These values reflect better characteristics of the solution. These values are also linked to the emulsion’s stability of 88.16 ± 2.37%. Other rheological parameters of the E10 emulsion are shown, including viscosity value (311.58 ± 4.78 cp), acidity (6.02 ± 0.34 g citric/L), and the pH value 6.34 ± 0.54.

### Scanning electron microscope of composite

The scanning electron microscope captures and reflects the composite composition’s oil and POE distribution. Figure 3.1 shows composite materials distributed in geometrical shapes to form caves and curving shapes that support the POE loading to composite materials. These curves also prevent the extract components from degradation until they do their functions. Adding oil to the composite material enhanced the extract stability in the composite. This characteristic is evident in Fig. 3.2, where the oil with low loading concentration shows the lock function of the curves loaded by the extracted content. While few amounts of jojoba oils were aggregated to form tiny droplets, most of the oil content formed a slime membrane around the extract in the composite cavities (Fig. 3.3). The nature of jojoba oil as a waxy-fat material assessed its multifunction role in the composite. The presence of oil may play several functions in grazing the composite cavity, supporting the extract functionality, and protecting loaded components of the POE.

**Fig. 3:**
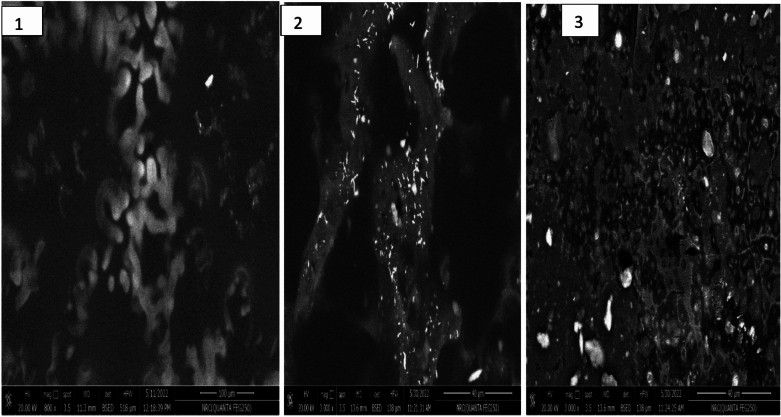
Scanning electron microscope of (**1**) control nano-composite, (**2**) oil-loaded composite, and (**3**) oil-POE-loaded composite.

The POE release from the composite content will be controlled by composite cavity binding and the oil waxing effect. This characteristic can improve the POE efficiency for its antimicrobial potency. Also, the presence of oil in the composite structure can support its elasticity and flexibility. This property facilitates the composite application as a spraying emulsion or a high antimicrobial activity efficiency packaging composite.

### Molecular docking study

The molecular docking approach was applied in the current study to understand better how the phytochemicals in POE, JOE, and the polymers under investigation provide antibacterial and anti-flatoxigenic activities. They analyzed the binding free energies of the various constituents present in the composites, including phenols, flavonoids, fatty acids, and polymers as ligands. The antibacterial properties of these ligands were revealed through docking into vital bacterial enzymes involved in the biosynthesis and repair of cell walls, proteins, and nucleic acids (PDB IDs: 1JZQ, 1KZN, 2VEG, 2ZDQ, 3RAE, 3SRW, and 3UDI). The Table displays the best poses obtained from the molecular docking analyses, revealing the binding affinities of each ligand with the different bacterial enzymes and consequently, the antibacterial activity of these ligands. A lower ∆G indicates a more vital interaction between the receptor and the ligands. Among the investigated ligands, flavonoids displayed higher binding affinities with high docking scores, ranging from -6.6 to -11.1 kcal/mol, which means a potential antibacterial activity, followed by phenolic acids with binding energies between -4.9 and -9.8 kcal/mol towards the different bacterial enzymes (Table 9).

**Table 9:** Binding free-energy values are calculated through the molecular docking of the polymers, POE, and JOE major constituents and the bacterial key metabolic enzymes as receptors.

Ligand	Binding Free Energy ΔG (- kcal/mol)						
	1JZQ *	1KZN	2VEG	2ZDQ	3RAE	3SRW	3UDI
*Phenolic acids*							
Ellagic	-8.2	-8.3	-7.8	-9.8	-8.0	-8.7	-7.9
Cinnamic	-5.7	-6.0	-5.3	-7.5	-5.7	-5.8	-5.4
Caffeic	-6.1	-6.2	-5.7	-8.0	-6.2	-6.4	-6.1
Gallic	-5.7	-5.9	-5.5	-6.8	-5.7	-5.7	-5.9
*p-*Coumaric	-5.7	-6.0	-5.2	-7.8	-5.6	-6.1	-5.8
*p-*Hydroxybenzoic	-5.3	-5.7	-4.9	-6.7	-5.4	-5.4	-5.4
Protocatechuic	-5.6	-6.1	-5.5	-6.9	-5.5	-5.6	-5.6
Syringic	-5.6	-5.4	-4.9	-6.8	-6.1	-6.0	-5.3
Ferulic	-6.0	-6.3	-5.3	-7.6	-6.5	-6.5	-6.0
Vanillic	-5.5	-6.0	-5.1	-6.9	-5.6	-5.7	-5.6
*Flavonoids*							
Naringenin	-7.9	-8.2	-7.3	-8.9	-7.9	-8.6	-7.7
Apigenin	-7.9	-8.4	-7.1	-8.8	-8.1	-8.4	-7.9
Luteolin	-8.1	-8.9	-7.6	-9.1	-8.1	-8.9	-8.3
Rutin	-9.9	-7.4	-8.6	-8.4	-8.3	-9.0	-9.8
Hesperidin	-9.7	-9.8	-8.2	-10.4	-9.0	-11.1	-9.5
Kaempferol	-8.2	-8.0	-6.6	-8.8	-8.0	-8.7	-8.3
Quercetin	-8.3	-8.3	-7.0	-8.9	-8.0	-9.0	-8.5
Catechin	-7.9	-8.4	-7.6	-9.3	-7.6	-8.5	-8.2
*Fatty acids*							
Erucic	-5.3	-5.7	-5.0	-7.0	-5.5	-6.2	-5.1
Gondoic	-5.7	-6.2	-5.1	-7.2	-5.2	-6.0	-5.3
Oleic	-5.7	-6.0	-5.3	-7.3	-5.9	-6.1	-5.3
*Polymers*							
Shellac	-7.1	-6.1	-6.2	-6.0	-6.5	-7.6	-6.9
CMC	-5.2	-4.6	-4.7	-5.7	-5.0	-5.1	-5.7

*Protein PDB ID; 1JZQ: isoleucyl- tRNA synthetase, 1KZN: DNA gyrase, 2VEG: dihydropteroate synthase, 2ZDQ: D-alanine: D-alanine ligase, 3RAE: IV topoisomerase, 3SRW: dihydrofolate reductase, and 3UDI: penicillin-binding protein 1a.

Ellagic acid showed the highest binding free energy (-7.8 to -9.8 kcal/mol) among phenolic acids due to its unique structure resulting from the dimerization of gallic acid through oxidative aromatic coupling. Most flavonoids in the study belong to flavones, except rutin and hesperidin. Rutin is quercetin with glucose and rhamnose sugar groups bound to it, and hesperidin is a disaccharide derivative of hesperetin. The binding affinity of rutin ranges from -7.4 to -9.9 kcal/mol, while that of hesperidin is between -8.2 to -11.1 kcal/mol. Fatty acids examined as ligands showed comparable results against all the enzymes associated with bactericidal/bacteriostatic effects with binding energies ranging from -5 to -7.3 kcal/mol, which revealed lower antibacterial activity compared to flavonoids or phenolics. Shellac showed higher affinities toward the bacterial enzymes tested as receptors and therefore higher antibacterial activity (between -6 and -7.6 kcal/mol) compared to CMS (from -4.6 to -5.7 kcal/mol) due to structure differences which affected the interactions and bonds formed with the receptors (Table 9). The previous findings agreed with the *in-vitro* results obtained in the current study, where POE showed the highest antibacterial activity, in contrast to JOE, which exhibited the lowest compared to the composites formulated.

Similar results to the above findings were obtained for the affinities towards different enzymes associated with aflatoxins biosynthesis such as aflatoxin- synthase (B8N0E8), cytochrome P450 monooxygenase (A0A1R3RGJ7), and halogenase (A0A1R3RGJ2), which revealed the antiaflatoxegnic properties of the ligands. Flavonoids were the most potential anti-aflatoxegenic, followed by phenolic acids, while fatty acids were the least. Hesperidin and rutin showed the highest affinities towards non-ribosomal peptide synthase (-10.7 kcal/mol) and cytochrome P450 monooxygenase (-9.6 kcal/mol), as shown in Table-. Meanwhile, ellagic acid and the four enzymes showed comparable results as the most potential phenolic acid, with binding free energies ranging from -7.8 to -9.1 kcal/mol. Erucic and gondoic acids showed better affinities towards halogenase and non-ribosomal peptide synthase than oleic acid. However, shellac had a higher affinity towards all the enzymes used as receptors, especially polyketide synthase (-7.7 kcal/mol), compared to CMC (Table 10). These findings align with the in-vitro results of antifungal and anti-aflatoxigenic properties. POE recorded the highest activity, and JOE was the least compared to composites containing both extracts.

**Table 10:** Binding free-energy values are calculated through the molecular docking of the significant phytochemicals of polymers, POE, and JOE major constituents and aflatoxins producing enzymes as receptors.

Ligand	Binding Free Energy ΔG (-kcal/mol)			
	Polyketide synthase	Halogenase	Cytochrome P450 monooxygenase	Non-ribosomal peptide synthase
*Phenolic acids*				
Ellagic	-7.8	-8.1	-8.8	-9.1
Cinnamic	-6.4	-6.8	-6.5	-6.6
Caffeic	-7.4	-6.7	-6.0	-7.0
Gallic	-7.2	-6.5	-5.9	-6.7
*p-*Coumaric	-6.7	-7.0	-6.1	-6.7
*p-*Hydroxybenzoic	-6.7	-5.7	-5.9	-6.5
Protocatechuic	-7.0	-6.3	-6.1	-6.6
Syringic	-6.3	-6.8	-6.2	-6.2
Ferulic	-6.6	-6.9	-6.3	-6.6
Vanillic	-6.8	-6.2	-6.2	-6.5
*Flavonoids*				
Naringenin	-7.6	-8.2	-8.3	-8.6
Apigenin	-7.8	-8.1	-8.3	-8.6
Luteolin	-8.0	-8.6	-8.5	-9.0
Rutin	-9.3	-8.4	-9.6	-8.8
Hesperidin	-9.3	-9.3	-9.3	-10.7
Kaempferol	-7.8	-7.8	-8.2	-8.3
Quercetin	-8.1	-8.2	-8.3	-8.5
Catechin	-7.7	-7.8	-8.2	-8.6
*Fatty acids*				
Erucic	-6.1	-7.4	-6.8	-7.0
Gondoic	-6.0	-7.1	-6.8	-7.3
Oleic	-6.4	-6.0	-6.7	-6.4
*Polymers*				
Shellac	-7.7	-7.1	-7.2	-7.5
CMC	-5.8	-5.4	-5.1	-5.4

polyketide synthase (A0A1R3RGK0), non-ribosomal peptide synthase (B8N0E8), cytochrome P450 monooxygenase (A0A1R3RGJ7), and halogenase (A0A1R3RGJ2).

**Fig. 4:**
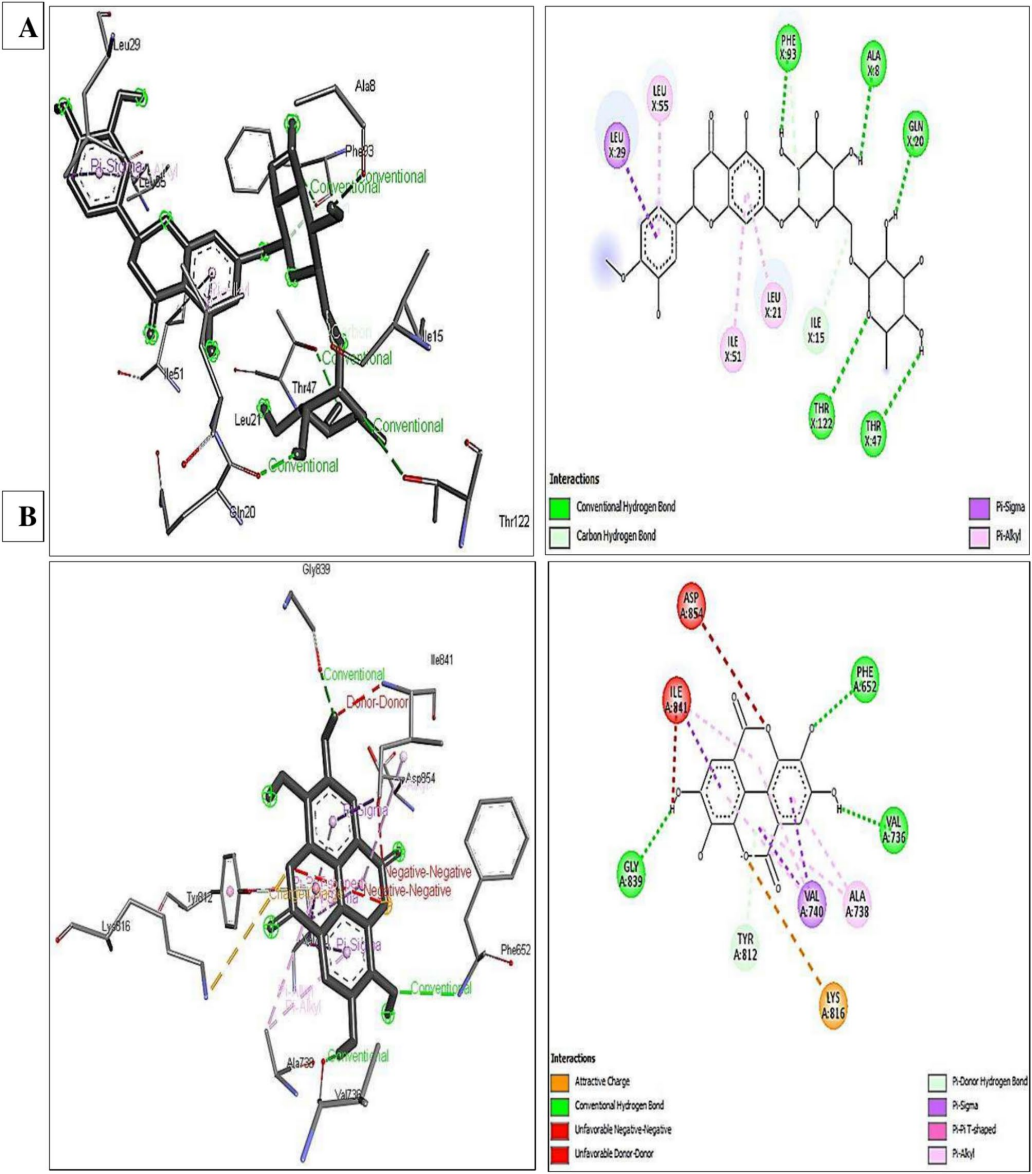
Molecular docking simulation for the Interactions between (**A**) hesperidin with 3srw and the interaction between (**B**) ellagic acid with B8N0E8.

Figure 4A and B demonstrate the interaction between hesperidin and ellagic acid with the crystal structure of dihydrofolate reductase (PDB:3SRW) and non-ribosomal peptide synthase (B8N0E8). These figures reveal that hesperidin and ellagic acid have the highest docking scores among flavonoids and phenolic acids which reflects the higher antibacterial and anti-aflatoxegenic properties of these ligands. Due to conventional hydrogen bonding, hesperidin has a higher binding affinity with 3srw (-11.1 kcal/mol). The oxygen of the hydroxyl groups from the legend acts as an H-donor and bonds with the O-of hydroxyl and carbonyl groups (H-acceptor) of PHE A:93, THR A:47, GLN A:20, and ALA A:9. Additionally, THR A:122 acts as a donor to the legend pyranose oxygen. C-H bonds were also noticed between the C- of pyranose rings of the hesperidin and the carbonyl oxygen of PHE A:93 and ILE A:15 (Figure–A). Hydrogen bonds are the main intermolecular interactions in biological complexes, and the free energy associated with hydrogen bonding can vary from -1.5 to -4.7 kcal/mol^89^.

In addition to hydrogen bonding, π-σ and π-alkyl hydrophobic interactions were observed between 3srw residues and hesperidin. The C-H of LEU A:29 contributed to the π-orbitals of the legend, and C-H⋯π-interactions can be classified as weak hydrogen bonds. Interactions of LEU A:21, ILE A:51, and LEU A:55 alkyl groups with aryl-containing legend through π-orbitals often expose their aromatic side chain to the binding site (Fig. 4A). Aromatic rings’ particular shape and electronic properties result in preferred interaction geometries. High-level ab initio calculations of dimer dissociation energies between benzene and ethane yield -1.8 kcal/mol with the sp3-hybridized ethane^90^. Hydrophobic interactions are the most common interactions in protein–ligand complexes, and an aliphatic carbon in the receptor and an aromatic carbon in the ligand create most interactions in this group. 76% of commercially available pharmaceuticals contain one or more aromatic rings, with the benzene ring system being the most common aromatic system^89^.

With a few conventional solid hydrogen bonds, a lower binding free energy could be observed between ellagic acid and non-ribosomal peptide synthase (B8N0E8) with -9.1 kcal/mol. These conventional H-bond could be observed between the N- of PHE A:652 and O- of the legend as H-donors and the hydroxyl and carbonyl groups of the legend, GLY A:839, and VAL A:736 (Fig. 4B). A weak hydrogen bond in the shape of π-donor and π-σ hydrophobic interactions from the O- of TYR A:812 and C-H of VAL A:740 and ILE A:840 to the π-orbitals of the legend. In addition, π-alkyl hydrophobic interactions were conducted from the π-orbitals of the legend to the alkyl of ALA A: 738, VAL A:740, and ILE A:841 (Fig. 4B). Bissantz et al.^86^ stated that the T-shaped edge-to-face and the parallel-displaced stacking arrangement are the most common interactions between two π systems. For instance, TYR A: 812 and legend’s π-orbitals exhibited a π-π T-shaped interaction. A positively charged nitrogen is a highly electronegative substituent, resulting in a robust, attractive interaction. The interaction between a positively charged nitrogen, such as in LYS A:816, and a negatively charged oxygen, such as the pyranose oxygen of the legend, is explained as among the most frequent interaction types^89^. The number of salt bridge interactions with positive nitrogen from the protein and negative oxygen from the ligand was twice as high as the opposite.

### Bivariate correlation regarding the composite film content

The most robust and noteworthy associations found are between antioxidant activity and the decrease of the AFB_1_, phenolic chemicals, and the reduction of aflatoxin. These findings indicate that composite film’s antioxidant potency and phenolic content can reduce aflatoxin contamination. The findings suggest that the composite film’s phenolic content and antioxidant activity significantly contribute to the antifungal capabilities of the composite films, especially in terms of lowering the presence of hazardous chemicals like aflatoxins. These observations may provide valuable information for the future studies, especially in creating food packaging or coatings that use these molecules to improve safety and efficacy. Table (11) displays a bivariate correlation analysis among the properties of the composite film, the concentration of phenolic compounds, their antioxidant activity, and their effectiveness in reducing aflatoxin B1 and exhibiting fungicidal properties. The statistical relationships, presented in (Table 11), elucidate the potential functional characteristics of food packaging materials as influenced by their phenolic content. A significant positive correlation was recorded between higher phenolic content and the increase in antioxidant activity. This relationship is fundamental, as phenolic compounds are known for their antioxidant properties, which contribute to food preservation. A correlation was reported between the phenolic content of food packaging composites and toxigenic fungi reduction; this positive correlation hints at a potential connection, indicating that increased phenolic content might still be beneficial for reducing the fungal presence of food materials coated with the composites, as mentioned.

**Table 11:** Bivariate correlation between the composite film properties and its content of phenolic compounds, antioxidant potency, POE, and Jo; also its aflatoxin B1-reduction impact and fungal reduction properties impact.

Relation	Correlation coefficient	P (2-tailed)
PC & AA	0.36*	0.021
AA & TFG-red	0.54*	0.144
PC & TFG-red	0.44*	0.227
AA & AF-red	0.96**	0.000
PC & AF-red	0.89*	0.008
POE & MP-red	0.37*	0.241
JO & MP-red	– 0.31**	0.205
CMC & MP	0.48**	0.048
Sh & MP	– 0.22*	0.058
POE & AFE	0.94*	0.001
JO & AFE	0.55**	0.071

(*) Correlation is significant at the 0.05 level (2-tailed);(**) Correlation is significant at the 0.01 level (2-tailed).

PC: phenolic compounds; AA: antioxidant activity; TFG-red: toxigenic fungal reduction; AF-red:aflatoxin B_1_ reduction; MP-red: mechanical properties reduction; CMC:carboxymethyl cellulose; Sh: shellac; AFE:antifungal efficiency

The relationship between the antioxidant activity of film composites and the AFB_1_ Reduction reflects a powerful positive correlation, with an R-value of 0.96 (p = 0.000), indicating that higher antioxidant activity is closely linked to the reduction of AFB_1_, and that means more efficiency for performed composites to preserve food against contamination and cross contamination by mycotoxin during its storage. Again, this suggests that the composite films’ antioxidant properties significantly contribute to minimizing aflatoxin contamination, highlighting their important role in food safety. Moreover, a correlation between phenolic content and antifungal efficiency (POE & AFE: 0.94, p = 0.001; JO & AFE: 0.55, p = 0.071) indicates a strong relationship, where phenolic compounds contribute significantly to the antifungal properties of the films.

The R-value between Phenolic Compounds and AFB1 Reduction is 0.89 (p = 0.008), which also indicates a strong positive correlation. This further reinforces the idea that phenolic compounds boost aflatoxin reduction, potentially offering a dual benefit of antioxidant and antifungal properties.

The presence of mechanical properties being influenced by other components like CMC also highlights the importance of composite material formulation in achieving optimal packaging characteristics without compromising functionality. The correlation between phenolic compounds and the impact on mechanical properties reduction was recorded as moderate at r = 0.37 (p = 0.241), hinting at some interaction but not definitive at the 0.05 significance level. The relationship between the films’ composition (such as the CMC or Sh) and the mechanical characteristics recorded by varied effects. The CMC exhibited a significant correlation (0.48, p = 0.048), while shellac showed a weaker negative correlation (–0.22, p = 0.058).

The identified correlations indicate that the inclusion of phenolic compounds in composite food packaging materials can improve both antioxidant capacity and efficacy against harmful fungi and aflatoxins. The correlation between antioxidant activity and aflatoxin reduction is significant, indicating that the creation of packaging with elevated phenolic content may enhance food safety and shelf life.

It is worth noting that pomegranate peel extracts are recognized for their antifungal activity due to their phenolic and flavonoid content. For instance, punicalagin demonstrated efficacy against the conidial and hyphal phases of *Trichophyton mentagrophytes, T. rubrum*, *Microsporum canis*, and *M. gypseum*^75^. The elevated gallic acid concentration effectively eradicates *T. rubrum*^91^. Furthermore, benzoic acid and its derivatives present in the peel extract inhibited *A. flavus* mycelial growth by obstructing the fungal mitotic exit network and cytokinesis, as well as by suppressing the biosynthesis of the AFB_1_ and AFB_2_^92^. Cinnamic acid and its derivatives inhibit the enzymatic activity of CYP53A15, demonstrating the extract’s antifungal properties^93^. Research indicates that ellagitannins extracted from botanical sources, such as pomegranate peels, exhibit inhibitory effects against the phytopathogenic fungi *Alternaria alternata, Fusarium oxysporum f. sp. lycopersici, Colletotrichum gloeosporioides*, and *Rhizoctonia solani*^94^. This evidence could illustrate the correlation recorded between the phenolic content of film composites, regarding their pomegranate content, and the impact on toxigenic fungi reduction of the growth and with the aflatoxin secretion reduction.

Moreover, numerous flavonoids demonstrate potential as economical agents for combating fungal infections. They inhibit fungal growth by disrupting the plasma membrane, leading to mitochondrial dysfunction and interfering with cell wall formation, division, and protein synthesis. Furthermore, these flavonoids may improve the effectiveness of traditional medications, rendering them significant in the advancement of new treatments for fungal pathogens^95^. These points illustrate the correlation between the phenolic compound of film composites (based on pomegranate) and their potency to preserve food materials by their coating performing application in food production.

In summary, the most robust and noteworthy associations found are between antioxidant activity and the decrease of aflatoxin B_1_, phenolic chemicals, and the reduction of aflatoxin. These findings indicate that composite film’s antioxidant potency and phenolic content can reduce aflatoxin contamination. The findings suggest that the composite film’s phenolic content and antioxidant activity significantly contribute to the antifungal capabilities of the composite films, especially in terms of lowering the presence of hazardous chemicals like aflatoxins. These observations may provide valuable information for future studies, especially in creating food packaging or coatings that use these molecules to improve safety and efficacy.

## Conclusion

CMC/shellac composites were used for food packaging and compared to their POE/JOE loading formulas at varied concentrations. Emulsions were examined before drying to assess composite material activity. The POE has high phenolic compound (phenolic acids and flavonoids) content and antioxidant activity. Composites of films E10–E16 have substantial phenolic and flavonoid content associated with their POE content. Composites that have more phenolic are E12, and flavonoid concentration is highest in E15. High CMC content, moderate shellac, and JO: POE loading increase mechanical characteristics. AP enhances physical attributes best in E13, E10, E8, and E7. The composite films with optimum burst strength were E6, followed by E11. The composites’ unique anti-mycotoxigenic effect reduces aflatoxin levels while limiting toxigenic fungal growth using a simulated coating experiment of corn cereals. Molecular docking simulation shows the effectiveness of composite ingredients and minor components in terms of antibacterial and anti-mycotoxigenic properties. Results suggested E10 (0.3/1; POE/JOE) as the optimum food packaging composite. It is considered a unique cereal coating or packaging material that saves cereal and cereal-based food against toxigenic fungi contamination and their mycotoxins. Future investigations are significant in discovering other distinguished composite materials loaded with antimicrobial plant extract for food shelf life extension of packaged fruits, vegetables, and other natural items.

## Data Availability

Data for the current study are available from the corresponding author upon reasonable request.
